# Cotton Fiber Cell Walls of *Gossypium hirsutum* and *Gossypium barbadense* Have Differences Related to Loosely-Bound Xyloglucan

**DOI:** 10.1371/journal.pone.0056315

**Published:** 2013-02-14

**Authors:** Utku Avci, Sivakumar Pattathil, Bir Singh, Virginia L. Brown, Michael G. Hahn, Candace H. Haigler

**Affiliations:** 1 Complex Carbohydrate Research Center, University of Georgia, Athens, Georgia, United States of America; 2 Department of Crop Science, North Carolina State University, Raleigh, North Carolina, United States of America; 3 Department of Plant Biology, University of Georgia, Athens, Georgia, United States of America; 4 Department of Plant Biology, North Carolina State University, Raleigh, North Carolina, United States of America; New Mexico State University, United States of America

## Abstract

Cotton fiber is an important natural textile fiber due to its exceptional length and thickness. These properties arise largely through primary and secondary cell wall synthesis. The cotton fiber of commerce is a cellulosic secondary wall surrounded by a thin cuticulated primary wall, but there were only sparse details available about the polysaccharides in the fiber cell wall of any cotton species. In addition, *Gossypium hirsutum* (*Gh*) fiber was known to have an adhesive cotton fiber middle lamella (CFML) that joins adjacent fibers into tissue-like bundles, but it was unknown whether a CFML existed in other commercially important cotton fibers. We compared the cell wall chemistry over the time course of fiber development in *Gh* and *Gossypium barbadense* (*Gb*), the two most important commercial cotton species, when plants were grown in parallel in a highly controlled greenhouse. Under these growing conditions, the rate of early fiber elongation and the time of onset of secondary wall deposition were similar in fibers of the two species, but as expected the *Gb* fiber had a prolonged elongation period and developed higher quality compared to *Gh* fiber. The *Gb* fibers had a CFML, but it was not directly required for fiber elongation because *Gb* fiber continued to elongate rapidly after CFML hydrolysis. For both species, fiber at seven ages was extracted with four increasingly strong solvents, followed by analysis of cell wall matrix polysaccharide epitopes using antibody-based Glycome Profiling. Together with immunohistochemistry of fiber cross-sections, the data show that the CFML of *Gb* fiber contained lower levels of xyloglucan compared to *Gh* fiber. Xyloglucan endo-hydrolase activity was also higher in *Gb* fiber. In general, the data provide a rich picture of the similarities and differences in the cell wall structure of the two most important commercial cotton species.

## Introduction

Cotton fiber harvested from *Gossypium* species is the world’s most important renewable textile fiber. These single-celled fibers are highly elongated and thickened seed epidermal cells, and their useful properties depend on cell walls deposited during a staged cellular differentiation program lasting about seven weeks. Plant cell walls include fibrillar cellulose and various other polysaccharides in a surrounding matrix, e.g. xyloglucan (XG) and pectins including homogalacturonan (HG), plus variable percentages of protein and sometimes lignin. In cotton fiber, a thin primary cell wall is synthesized while the fiber achieves its extraordinary length of ∼2.5 to 3.5 cm. During the transition to secondary wall cellulose synthesis, primary wall remodeling occurs and a thin intermediary cell wall “winding” layer (analogous to the S1 layer in wood fiber) is deposited. The transition stage ends when secondary wall thickening begins via the deposition of nearly pure cellulose (an unusual feature of cotton fibers). At harvest, the cotton fiber is composed of a thick cellulosic secondary wall that is surrounded by a ∼200 nm thick cuticulated primary wall on the fiber perimeter [1]–[4].

Despite similarities in these basic cotton fiber characteristics, there are important differences in the fiber produced by the two commercial allotetraploid species, *Gossypium hirsutum* (hereafter *Gh*) and *Gossypium barbadense (Gb)*. Polyploidization about 1–2 million years ago unified ancestral diploid A and D genomes to form the progenitors of modern allotetraploid *Gh* and *Gb* cotton, followed by independent evolution and then domestication of the two species [5]–[6]. *G. hirsutum*, or Upland cotton, accounts for the majority of current cotton fiber production because of its high yield and wider environmental adaptability. However, *Gb* cotton is grown in selected environments because its fiber (called Pima or Egyptian cotton) is longer, stronger, and finer (having less mass per unit length), which makes it preferred for spinning the stronger and silkier yarns that can be woven into luxury cotton clothing. A major goal of modern cotton breeding and biotechnology is to determine the controls of these superior fiber properties in *Gb*, followed by selective transfer of improved fiber traits to *Gh* without compromising its high yield and environmental adaptability.

Under suitable growing conditions, *Gb* fiber has a prolonged elongation phase that generates its longer fiber compared to *Gh* [7]–[9]. Similarly, finer fiber in *Gb* correlates with less cellulose per unit of fiber length ([10]; see also the results of this study). Recently, transcriptomics, analyses of recombinant inbred lines, and determining expression quantitative trait loci (eQTL) in interspecific backcrossed populations have provided clues about genetic differences that may result in higher quality *Gb* fiber [11]–[14]. Differences in cellular processes are also being discovered between the two species. For example, the duration of plasmodesmatal closing at the base of the fiber was much longer in *Gb* compared to *Gh*, which was predicted to allow high turgor to persist longer in *Gb* fiber as a driver of elongation [9]. Higher vacuolar invertase gene expression levels, vacuolar invertase activity, and hexoses, along with a somewhat faster fiber elongation rate, were observed in one *Gb* cultivar compared to *Gh* [15]. Similar observations were made for phospho*enol*pyruvate carboxylase, a key malate biosynthetic enzyme, which may promote fiber elongation through effects on turgor, lipid synthesis, or other processes [16].

Although other studies have implicated differences related to genetic control of cell wall synthesis in *Gh* and *Gb* fiber [11]–[14], there was previously little evidence about how the fiber cell wall chemistry of these two commercial cotton species compared. Addressing this deficiency is especially important in light of recently discovered unexpected features of cotton fiber cell walls. For example, *Gh* fibers are joined together during elongation by a special outer layer of the primary wall called the cotton fiber middle lamella (CFML) [3]. The CFML facilitates the formation of tissue-like fiber bundles, which become organized into a packet around each seed. This orderly fiber packing, in turn, likely facilitates the extreme elongation of >100,000 fibers within a confined space inside each locule (or carpel) of the cotton boll (or fruit). The CFML of *Gh* fiber contained epitopes typically found in primary cell matrix polysaccharides, including XG with some fucosylated epitopes, and HG with relatively low or no esterification. (See [17] for details on XG structure and [18] for explanation of HG structure.) The CFML breaks down at the onset of secondary wall deposition in *Gh* fiber, whereas the inner primary wall layer surrounding the protoplast persists. Corresponding changes in the chemistry of *Gh* fibers were shown by analysis of cell wall polymer sugars and by use of thirteen specific probes to detect polymer epitopes in dot blots of fiber wall extracts [3]. This study illustrated the potential of using specific probes for epitopes in cell wall polysaccharides, such as monoclonal antibodies and other carbohydrate binding proteins, to generate information about cell wall chemistry relatively rapidly and efficiently [19].

It was previously unknown whether the fiber of *Gb* had a CFML, and a large-scale comparison of cell wall structure in *Gh* vs. *Gb* cotton fiber was not available. To address these deficiencies, the research reported here took advantage of large scale Glycome Profiling [20] using a toolkit of 149 monoclonal antibodies directed against non-cellulosic carbohydrate epitopes within the cell wall matrix [21]. Cotton fiber cell walls from two species at seven stages of development were extracted to yield four cell wall polymer fractions per sample, which were then analyzed using Glycome Profiling. Fluorescence immunohistochemistry of fiber cross-sections was carried out in parallel, showing differences in the fiber of the two species related to loosely bound XG in the CFML. Enzymatic assay of the potential to degrade XG also showed a difference between the two species. Collectively the results reveal similarities in the fiber walls of both species, as well as XG-related differences that can be investigated in future work as possible determinants of the superior quality characteristics of *Gb* fiber.

## Materials and Methods

### Growing Cotton and Measuring Fiber

Plants of *Gh* cv. Deltapine 90 (*Gh*) and *Gb* cv. Phytogen 800 (*Gb*) were grown in parallel between April to October in an air conditioned greenhouse with a 26/22°C diurnal temperature cycle and natural lighting. Flowers were tagged on the day of anthesis so that bolls of known DPA could be collected. Details of cotton growth conditions and procedures for hand-measurement of fiber growth parameters were described previously [3]. Automated fiber quality measurements were performed by Cotton Incorporated, Cary, NC using high volume instrumentation (HVI) and Advanced Fiber Information System (AFIS) analysis according to standard methods. Three random grab samples were taken from bulked, roller-ginned, fiber harvested in the summer of two years. Results from each sample were averaged, and significant differences between means for the two species were determined by T test.

### Expression Analysis of Fiber Development Marker Genes

RNA isolation and quantitative reverse transcription PCR were performed as described previously ([3]; see Table 1 for primers). Expression levels of each experimental gene were normalized to the value for the endogenous reference gene, *Gheif5* (validated previously; [22]), and the normalized values (ΔCt) were determined for at least three biological replications.

**Table 1:** Primers used in quantitative reverse transcription PCR.

Cotton Gene Target ID	Primer Name	Forward Primer Sequence	Reverse Primer Sequence
DT563086	expa4	ATATCGTGAAGGTGAGCGTGAA	GATTGACCAACCAAAACTGCATT
U58283	CESA1	TGGACTACCCGGTGGATAAGGT	CTTTCTTGCAAAGTCGGCTGTT
U58284	CESA2	CACTCGTGATCATCCTGGAATG	AAGTCGAGGCAGCTCTTTGC
CO496524	cesa7	GCCGGCAATCTGTTTACTTACC	GCTCGAGAATACCAGTTGCAAA
CO492947	eif5	GGTTGCCATTGTGCAAGGA	CCGTAGGTGAGCGTTAATCAGA

### Large-scale Glycome Profiling of Cotton Fiber Cell Wall Epitopes

ELISA using 149 monoclonal antibodies in 21 clades (or groups showing similar recognition patterns to a panel of plant cell wall polysaccharides; [21]; Table S1) and data analysis were carried out essentially as described previously [20], [23]–[24] except the chlorite and 4 M KOH post-chlorite extractions were omitted. The typical value for the water control (absorbance = 0.05) was subtracted from each average reading in the EXCEL sheet before the heat map was generated. Monoclonal antibodies were obtained as hybridoma cell culture supernatants from laboratory stocks at the Complex Carbohydrate Research Center. Antibodies in the CCRC, JIM, MAC, MH, and PN series are available from CarboSource Services (http://www.carbosource.net). The monoclonal antibody against ß-1,3-glucan, LAMP2HI2H7 (abbreviated here as LAMP), was obtained from Biosupplies Australia (#BS-400-2; http://www.biosupplies.com.au/; [25]). A list of antibodies used in this study is provided in Table S1, which also includes links to the Wall*Mab*DB database (http://www.wallmabdb.net) where additional information currently available for each antibody can be found.

To begin the Glycome Profiling, bolls of typical size from *Gh* and *Gb* at 14, 17, 19, 21, 24, 30, and 35 DPA were harvested and immediately cut open to remove the seed/fiber mass prior to snap freezing in liquid nitrogen and storage at −80°C. Under liquid nitrogen, fiber was separated from seeds and then ground to fine powder, followed by freeze-drying. Alcohol insoluble cell wall residue (AIR) was produced and then extracted with a series of increasingly strong solvents to remove cell wall polysaccharides sequentially according to their strength of binding within the cell wall [26]. First, lyophilized ground fiber was suspended in 80% (v/v) EtOH (20 ml, RT, overnight, shaking at 150 rpm) followed by centrifugation (6000 rpm, 15 min, RT), rinsing with vortexing in 10 ml 100% acetone, repeated centrifugation, resuspension in acetone, and drying (RT) until no acetone odor remained. For each sample, 2 or 3 biological replicates were processed to yield 200 to 750 mg of fiber AIR, with each replicate including fiber from up to ten bolls (or ∼240 seeds). The most bolls were required at 14 DPA when fiber mass was least.

Successive extractions for each sample (10 mg sample:1 ml solvent–typically 200 mg AIR:20 ml solvent) occurred starting with 50 mM ammonium oxalate (pH 5.0, RT, overnight, shaking at 150 rpm). After centrifugation (3000 rpm, 15 min, RT), the supernatant was saved at 4°C awaiting dialysis, and the pellet was successively extracted in 50 mM sodium carbonate (pH 10) containing 0.5% (w/v) sodium borohydride followed by 1 M KOH and then 4 M KOH, both containing 1.0% (w/v) sodium borohydride. (The pellets were washed with deionized water before sodium carbonate and 1 M KOH extractions). Supernatants from both KOH extractions were treated with 2–3 drops of n-octyl alcohol (to minimize frothing) and neutralized on ice with glacial acetic acid. The four supernatants were dialyzed for 48 h against de-ionized water (1∶60 sample:water ratio) with slow stirring (water replaced every 8 to 12 h).

The amount of sugar in the extracts was determined by the phenol-sulfuric acid method [27]–[28], and each ELISA well was loaded with an equal amount of sugar dissolved in water (50 µl/well from a 60 µg/mL solution). ELISA and data analysis were carried out as described previously [20]–[21]. In addition, an experiment was conducted to assess the potential effects on ELISA absorbance values of interference between cell wall polymers in complex mixtures, with details provided in the legend of Figure S1. Initially, the extracts from two independent fiber samples for each DPA in each species were assayed, and a third fiber sample was assayed in a few cases where the values did not initially match well. Finally, the values from two consistent biological replicates were averaged to yield the 8,232 ELISA absorbance values (Table S2).

### Immuno-dot-assay of Fiber Wall Material Extracted by Hot Acidic Water

CCRC-M1 and CCRC-M58 were used as probes in this assay. For both *Gh* and *Gb*, seeds with attached fibers were harvested at 10, 19, 24, and 30 DPA from bolls of three plants and assayed in parallel. Fibers were left attached to seeds in order to replicate the “fiber straightening” protocol commonly used for fiber measurements [3] and minimize extraction of intracellular components. Samples were heated in acidic water (15 ml 0.025 N HCl, pH 2.8 to 3.0, ∼100°C, 20 min), then filtered through Miracloth (http://www.emdchemicals.com). The supernatant was clarified by centrifugation (3000 rpm, 3 to 5 min) and adjusted to 15 ml with water containing 0.02% (w/v) NaN_3_ to compensate for evaporation. Standard methods of sugar and protein quantitation showed that there was 95–97% sugar and 3 to 5% protein in the extracts at different DPA (unpublished data), indicating that the hot acidic water mainly extracted cell wall carbohydrate materials from whole fibers still attached to seeds. Colorimetric immuno-dot-assays were performed as described [29]. The net intensity for each developed spot (subtracting the value for blanks) was determined (Kodak Image Station 440CF, http://carestream.com). Samples were diluted if necessary so that the intensity values were within a linear range established through using CCRC-M1 as a probe for a dilution series of 10 DPA fiber extract (unpublished data). Significant differences between means for intensity/µg sugar for the two species at each DPA were determined by T test.

### Microscopy and Immunohistochemistry

Fibers of *Gb* at 10, 17, 19, and 24 DPA were examined for the presence or absence of the CFML in the TEM as previously described [3]. For both *Gh* and *Gb*, cell wall epitopes in 250 nm thick cross-sections of 10, 17, and 24 DPA fibers were analyzed by fluorescence immunolabeling as described [21], [30] except that primary antibodies were diluted 1∶2 in blocking buffer. All images from a given antibody were captured at the same exposure time in an epifluorescence microscope (Nikon Eclipse 80i, the Nikon B-2E/C fluorescence filter set, DS-Ri1 camera, NIS-Elements Basic Research Software microscope; Nikon Instruments Inc., Melville, NY). For the high magnification fluorescence images, the Sharpen/Unsharp Mask filter was applied uniformly using Adobe Photoshop.

### Enzyme Activity Assays

Fiber (∼0.08 to 0.12 g fresh weight) was vortexed in 1.0 ml of 100 mM sodium acetate buffer (pH 4.0) containing 0.5 M NaCl, extracted (15 min, 4°C), and centrifuged to clarify and separate the supernatant (15,000 x *g*, 5 min then 10 min), which was stored on ice prior to assay. Activity of XG endo-hydrolase (XEH) was assayed as described with minor modification [31]. The reaction mixtures (200 µl total volume) contained 75 mM sodium acetate buffer (pH 6.0), 100 µl supernatant, 100 µg of tamarind XG (#P-XYGLN; Megazyme International, Wicklow, Ireland). Reactions with buffer replacing XG or the fiber extract provided the blank or the negative control, respectively. After incubation (30°C, 24 h), reactions were terminated by addition of 100 µl of 1 M HCl. Color development occurred (30 min, RT, dark) after addition of 800 µl 20% (w/v) Na_2_SO_4_ and 200 µl potassium tri-iodide reagent [1.0% KI (w/v) and 0.5% iodine (w/v) in water], including samples with 10 to 100 µg XG as standards. XEH activity in the fiber extracts (ng XG degraded h^−1^ mg protein^−1^) was calculated based on the decrease in the blue-green color (OD_620 nm_) that arises from iodine complexed with higher molecular weight XG (>10 kDa) [31].

## Results

### Comparison of Fiber Growth and Development Profiles in *G. hirsutum* and *G. barbadense*

**Figure 1:**
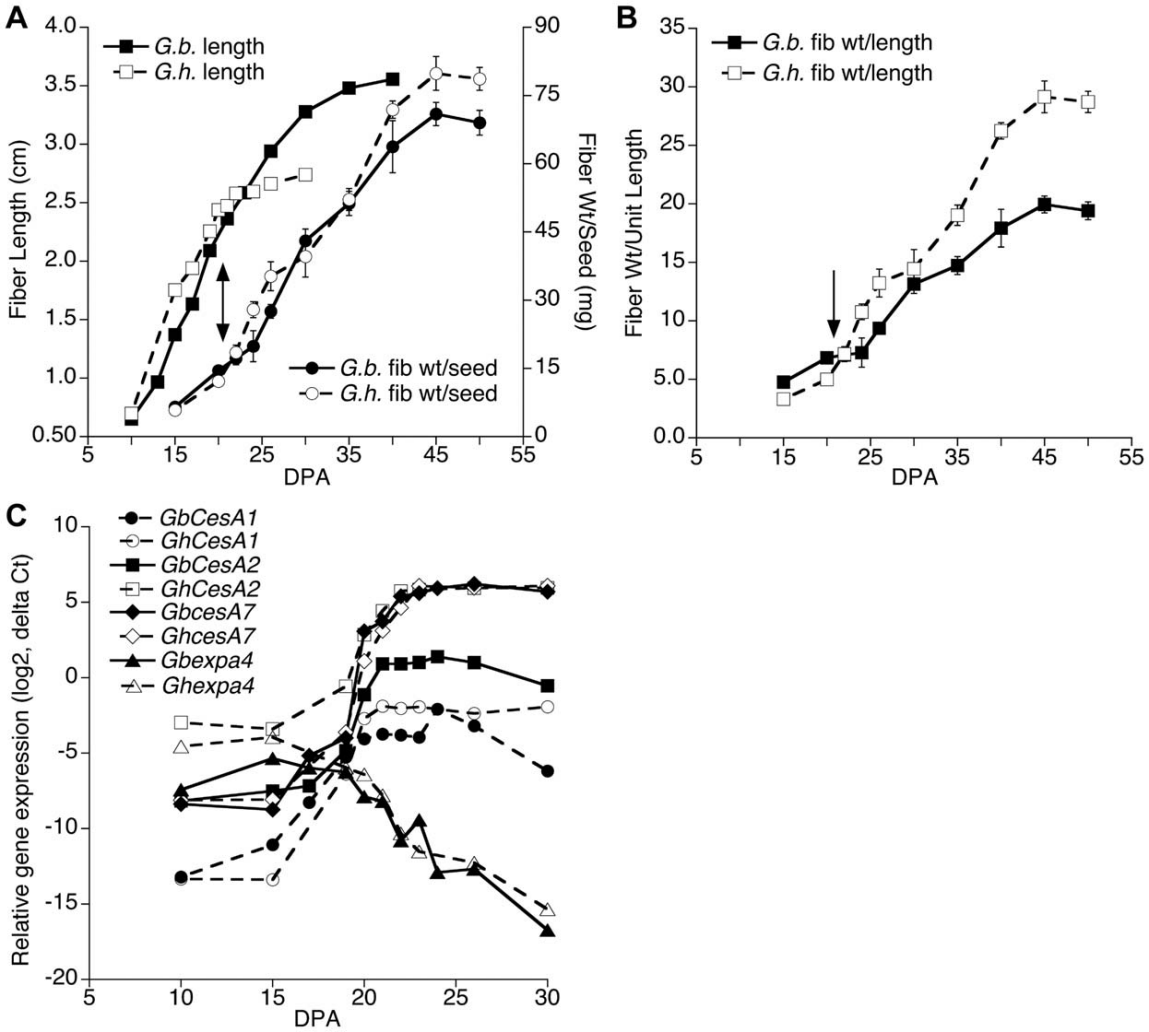
Fiber developmental progression between 10 to 50 DPA for *G. hirsutum* (*Gh*) and *G. barbadense* (*Gb*) documented by fiber measurements and gene expression. (**A, B**) Fiber length (hand measured) and fiber weight/seed (A) and the ratio of fiber weight to length (B). The timing of visible CFML degradation, which began ∼21 DPA near the onset of secondary wall deposition, is marked by arrows; see the data for *Gb* in Figure 2 and for *Gh* published previously (Singh et al., 2009). The data points (± SE) are the means of fiber measurements from 3 to 5 bolls of different plants. (**C**) Quantitative reverse transcription PCR data showing the developmental shift in gene expression prior to the onset of secondary wall cellulose synthesis at ∼22 DPA. For both *G. hirsutum* and *G. barbadense* fiber, the expression of three secondary wall-associated CESA genes began a sharp increase and the expression of an expansin isoforms began a sharp decline at ∼17 DPA. Each data point is the mean from 3 biological replicates. The fiber growth data for *Gh* up to 32 DPA (A) are republished from Figure S1 of [3] (http://www.plantphysiol.org, Copyright American Society of Plant Biologists).

*Gh* cv. Deltapine 90 and *Gb* cv. Phytogen 800, both elite modern cultivars, were grown in parallel in a 26/22°C greenhouse and fiber growth was measured (Figure 1 A and 1B) along with collection of fiber RNA for analysis of gene expression profiles (Figure 1C). The genes used as markers for fiber developmental stages included: (a) cellulose synthase genes (*CESAs*) that are apparently orthologous to the three CESA genes in *Arabidopsis thaliana* (*AtCESA4, AtCESA7, AtCESA8*) that are required for secondary wall thickening [32]; and (b) an expansin gene, representing a family of cell wall loosening proteins required for primary wall expansion [33]. Based on the results below and previous data for *Gh* fiber grown under the same conditions [3], the comparative stages of fiber development in the two species at the days assayed in these experiments are summarized in Table 2.

**Table 2:** Fiber differentiation processes on the DPA analyzed in these experiments.^a.^

DPA	Fiber developmental processes in *G. hirsutum*	Fiber developmental processes in *G. barbadense*
10	rapid elongation	rapid elongation
14	rapid elongation	rapid elongation
17	rapid elongation; early transition stage with shifting gene expression	rapid elongation; early transition stage with shifting gene expression
19 and 21	slowing elongation; the CFML degrades and the winding layer is synthesized during the transition to secondary wall deposition	**rapid elongation**; the CFML degrades and the winding layer is synthesized during the transition to secondary wall deposition
24	secondary wall synthesis	secondary wall synthesis; **rapid elongation**
30	secondary wall synthesis	secondary wall synthesis; **slowing elongation**
35	secondary wall synthesis	secondary wall synthesis

a Bold types highlights a major difference between species, specifically the prolonged elongation period for *G. barbadense* fiber.

The fiber of both species showed similar elongation rates between 10 to 22 days post anthesis (DPA) (Figure 1A). The slope of the linear regression lines (not shown) for fiber length vs. DPA (up to 22 DPA) was 0.16 for both *Gh* and *Gb,* respectively. At 22 DPA, elongation in *Gh* fiber essentially ended, but rapid elongation in *Gb* fiber continued for 8 more days until 30 DPA followed by slower elongation until at least 35 DPA.

The secondary wall *CESA* genes were up-regulated in *Gh* and *Gb* fiber beginning at ∼17 DPA (Figure 1C; see Table 1 for primers), indicating that genetic competence for wall thickening developed at nearly the same time in both species grown in parallel in a 26/22°C greenhouse. The expression level of an expansin gene began to decline at 17 DPA in both species, consistent with a major developmental shift occurring then. (Since expansins are a multi-gene family, other expansin isoforms may support the continued elongation of *Gb* fiber.) This transition stage of fiber development includes primary cell wall remodeling and the synthesis of the winding cell wall layer, and it ends at the time that nearly 100% cellulose synthesis begins (reviewed in [4]).

**Figure 2:**
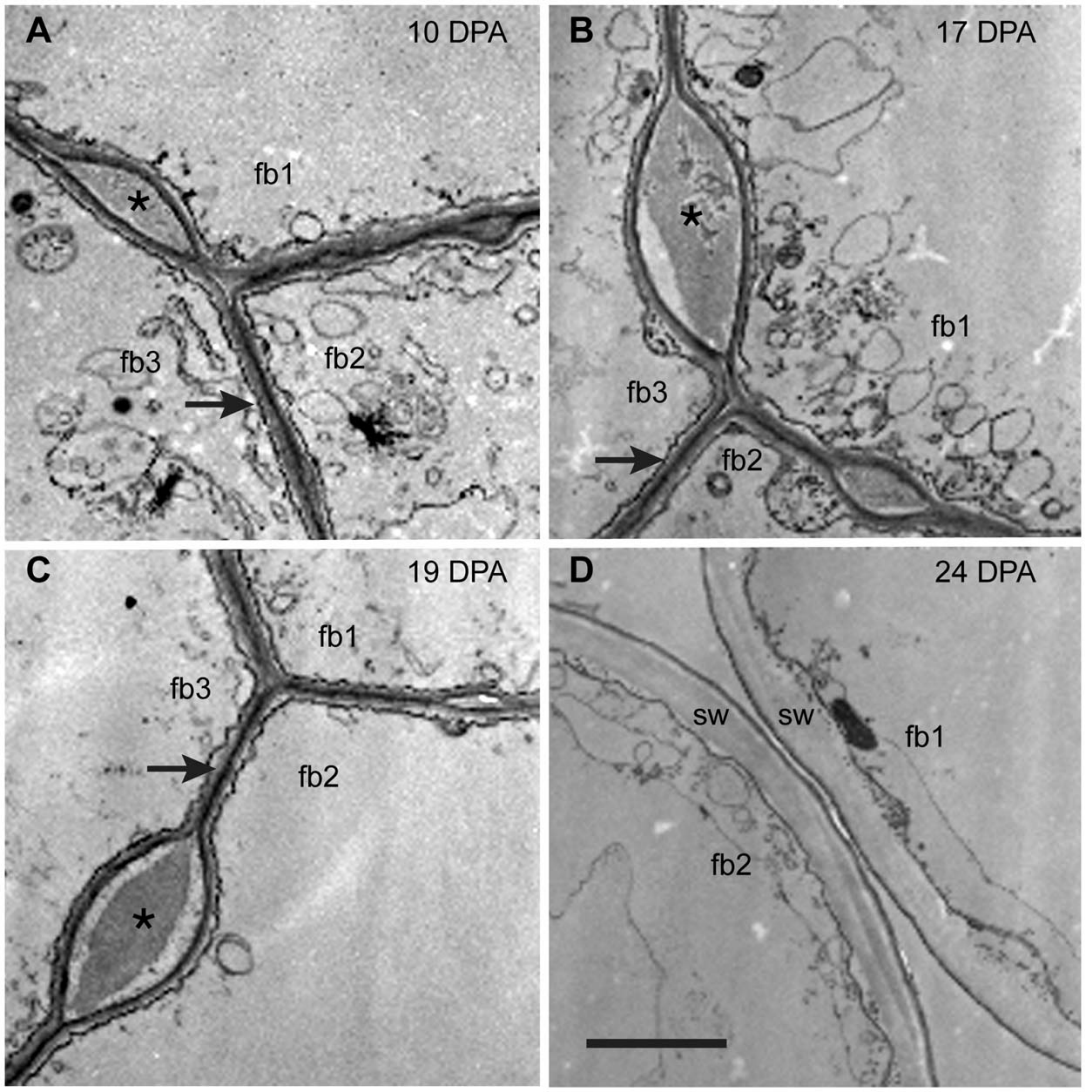
Transmission electron micrographs of cross-sectioned *G. barbadense* fiber at 10 to 24 DPA. The ‘fb#’ labels indicate 2 or 3 individual fibers in each view. (**A, B, C**) At 10 DPA, 17 DPA, and 19 DPA, adjacent fibers are joined together by the CFML, the outermost layer of the primary wall. In many regions, a thin continuous wall exists between adjacent fibers (arrows in A, B, C). However, there are also periodic bulges between fibers that are filled with CFML material (asterisks in A, B, C). (**D**) At 24 DPA during secondary wall (sw) deposition, the fibers have separated due to CFML degradation, leaving empty space between them. The 2 µm scale bar in D applies to all micrographs.

Near 22 DPA in both species, the expression level of the secondary wall CESA genes reached their maximum level (Figure 1C) and secondary wall cellulose synthesis began, as indicated by the increase in fiber weight per seed (Figure 1A) and thickened fiber walls observed by 24 DPA (Figure 2D). The slopes of the linear regression lines (not shown) for fiber weight/seed vs. DPA were similar for both species (2.6 or 2.4 for *Gh* or *Gb*, respectively, between 22 to 45 DPA). Therefore, a similar amount of fiber was deposited on each seed in both species, but fiber weight/seed does not reveal differences that occur within individual fibers. When the fiber weight/length ratio was calculated to normalize for different fiber lengths, increases indicative of cell wall thickening were also observed beginning ∼22 DPA in both species. Subsequently, the fiber weight/length ratio increased similarly from 22 to 30 DPA in both species (Figure 1B). In contrast, between 30 and 45 DPA the fiber weight/length ratio increased more slowly in *Gb* fiber. For the entire period of wall thickening (22 to 45 DPA), the slopes of the linear regression lines (not shown) for fiber weight/length vs. DPA were 0.93 for *Gh* and 0.59 for *Gb.* This difference is consistent with the lower final fineness of *Gb* fiber (see below).

### *G. barbadense* Fibers were Conjoined by a CFML Prior to Secondary Wall Synthesis

Images of cross-sectioned *Gb* fiber walls observed in the transmission electron microscope (TEM) showed that adjacent fibers were joined together at 10, 17, and 19 DPA during early elongation, but the cell wall material between adjacent fibers (the CFML) had broken down by 24 DPA when the secondary wall had started to form (Figure 2). As in *Gh* fiber [3], the cell walls of adjacent 10 to 19 DPA *Gb* fibers were often fused into one thin coherent wall (arrows, Figure 2A–2C), but bulged regions filled with cell wall material also occurred at intervals between fibers (asterisks, Figure 2A–2C) before the CFML was hydrolyzed. Whether or not the bulges in the CFML have particular functional significance is currently unknown. However, the bulges are inherent to the CFML early in fiber development and not indicative of CMFL breakdown at the transition stage (see 10 DPA *Gb* fiber in Figure 2A). A similar image for 10 DPA cryo-fixed *Gh* fiber was published previously (Figure 1G in [3]). Cotton fibers are well known to be difficult to fix chemically, and the cytoplasm of the fibers shown in Figure 2 was poorly preserved. Nonetheless, chemical fixation was usually preferred over cryo-fixation to examine the CFML because freezing often caused the CFML to crack [3].

### The Quality of *G. barbadense* Fiber was Higher than in *G. hirsutum*

As typically occurs under mutually suitable growing conditions, *Gb* fiber had higher quality compared to *Gh* fiber in a greenhouse with a 26/22°C temperature cycle (Table 3). All of the automated measurements of fiber length that are standard in the cotton industry (with outputs in inches) showed that *Gb* fiber was longer than *Gh* fiber. These measurements included: Upper half mean length, UHM; Length by weight, L(w); Upper quartile length by weight, UQL(w); Length by number, L(n); and Longest 5% of fiber by number, L5%(n). The automated length measurement UQL(w) obtained by AFIS (Table 3) was similar to the average fiber lengths obtained manually while generating the fiber growth profiles: 3.6 cm (1.4 in) and 2.7 cm (1.06 in) for *Gb* and *Gh* fiber, respectively. The strength of bundled fibers was also higher in *Gb* fiber (compare values for STR, g/tex, in Table 3). In contrast, *Gb* fiber had lower micronaire, which is a unitless parameter that depends on fiber perimeter and secondary wall thickness, as well as lower fineness (mTex, or mass per unit length). Maturity ratio, a reflection of fiber wall thickness relative to fiber perimeter, was similarly high in the two species (0.91 or 0.93 in *Gh* and *Gb*, respectively) (Table 3). Use of the fineness and maturity ratio values to calculate the outer fiber perimeter [34] generated a lower value for *Gb* (46.33 µm, corresponding to 14.75 µm cell diameter) compared to *Gh* (52.69 µm, corresponding to 16.78 µm cell diameter). The greater fineness of *Gb* fiber is explained by its smaller perimeter and lower cellulose accumulation per unit length.

**Table 3:** Fiber quality parameters determined by automated methods.^a^

Species/p value	MIC	STR [g/tex]	UHM [in]	L(w) [in]	UQL(w) [in]	L(n) [in]	L5% (n) [in]	Fineness [mTex]
*G. hirsutum*	4.7 (0.15)	30.37 (1.79)	1.15 (0.01)	1.04 (0.017)	1.20 (0.015)	0.883 (0.025)	1.37 (0.021)	176.3 (11.4)
*G. barbadense*	3.5 (0.15)	43.37 (0.55)	1.27 (0.02)	1.17 (0.006)	1.38 (0.010)	0.963 (0.021)	1.60 (0.015)	139.3 (3.5)
p-value	0.0007	0.003	0.0002	0.0028	0.0002	0.014	0.0002	0.02

a MIC, UHM, and STR were determined by HVI, and all other measurements derived from AFIS analysis. Mean values with (SD) are shown for fiber quality parameters that were significantly different in greenhouse-grown *G. hirsutum* cv. Deltapine 90 and *G. barbadense* cv. Phytogen 800 as determined by T test (n = 3 biological replicates, each including fiber from numerous bolls harvested over the whole plant).

### Glycome Profiling Revealed Similarities and Differences in Fiber Cell Wall Chemistry of *G. hirsutum* and *G. barbadense*

Glycome Profiling [20] employs enzyme-linked immunoabsorbent assay (ELISA) and a toolkit of 149 monoclonal antibodies [21] recognizing epitopes in plant cell wall matrix polymers in a high throughput approach to analyze cell wall composition. From fibers at seven DPA for both species, alcohol insoluble residue (AIR) was prepared and then extracted with four increasingly harsh solvents to remove loosely-bound followed by more tightly-bound polysaccharides [26]. Based on analysis of these four cell wall extracts per sample, Glycome Profiling identified various glycan-epitopes and revealed how tightly the glycans were bound into the fiber cell wall. The 149 monoclonal antibodies targeted multiple epitopes on all the major classes of plant cell wall matrix polysaccharides except rhamnogalacturonan II, and they fall into related clades based on their patterns of recognition of a diverse panel of isolated plant cell wall polysaccharides ([21]; see Table S1 for links to information on immunogens and antibody binding specificities). An experiment was conducted to assess the possibility of diminished epitope detection in the ELISA due to many co-mingled polysaccharides within a complex cell wall extract. Interference did not occur in most cases tested, but it was observed for several XG-directed antibodies, where a pectic arabinogalactan-rich extract suppressed XG antibody signals to about 50% at low XG amounts. In contrast, no suppression is observed at higher XG amounts (Figure S1). Although falsely reduced absorbance values cannot be ruled out, the possibility of overall misinterpretations is reduced by the use of several antibodies within one clade whenever possible, as is customary in Glycome Profiling, and cross-checking key conclusions with immunohistochemistry.

**Figure 3:**
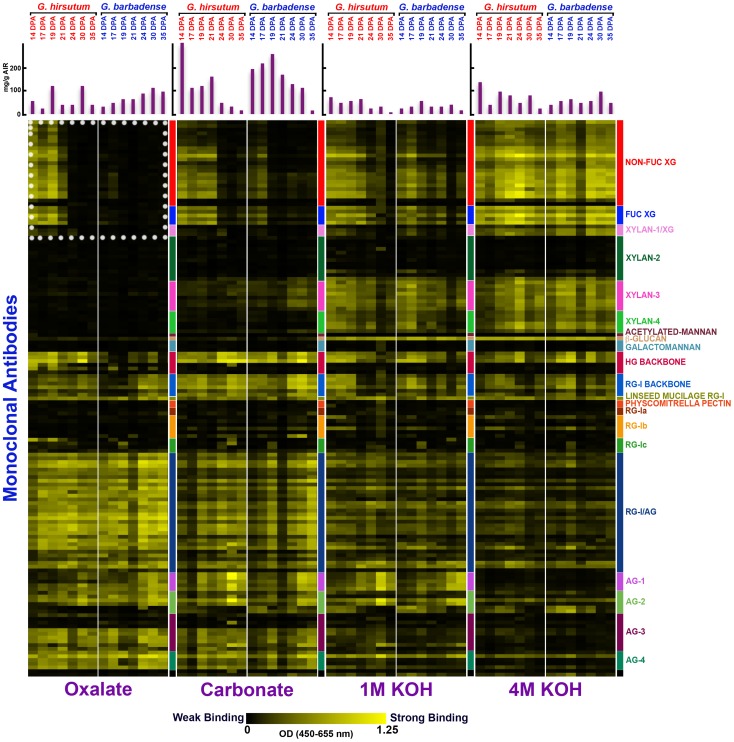
Glycome Profiling of sequential extracts prepared from multiple fiber samples representing major stages of cotton fiber development in *G. hirsutum* and *G. barbadense*. A vertical color-coded strip shows the clades of cell wall glycan-binding antibodies (as defined in [21]). Each successive cell wall solvent is shown on the bottom, with a split panel above each label showing the results for *Gh* and *Gb* fiber at 14 to 35 DPA. Absorbance values >0.10 typically are perceived as non-black. The bar graphs show the amount of cell wall material removed by each solvent from each sample (mg extracted/g AIR). The dotted white box outlines binding of XG-directed antibodies to the oxalate extracts, where the most striking differences between the two species were observed.

**Figure 4:**
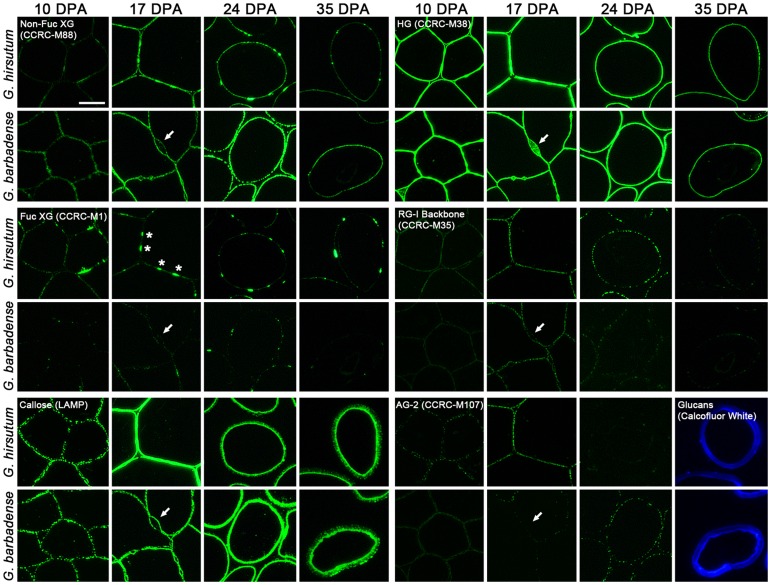
Fluorescence immunohistochemistry of *G. hirsutum* and *G. barbadense* fiber cross-sections at 10 to 35 DPA. The antibodies recognized epitopes found in XG (as shown by CCRC-M88 and CCRC-M1 labeling), ß-1,3-glucan or callose (LAMP), de-esterified HG (CCRC-M38), the RG-I backbone (CCRC-M35), and AG-2 (CCRC-M107). Calcofluor White, which recognizes cellulose and callose, stained the thickened fiber secondary wall at 35 DPA (images substituted for black panels in the lower right). The arrows indicate the location of a bulged region of the CFML in *Gb* fiber, which showed only HG in its interior. In contrast, HG and XG were in bulged regions of the CFML in *Gh* fiber (see the asterisks in the CCRC-M1 panel and [3]). Callose was detected within the thickening secondary wall at 35 DPA, whereas the XG and HG epitopes were only in the persistent primary wall on the fiber perimeter. The micrographs for each antibody were taken at the same exposure time. The 10 µm bar in the upper left panel applies to all micrographs.

The results of Glycome Profiling on fiber from *Gh* and *Gb* at 14 to 35 DPA are represented in a heat map (Figure 3; see Table S2 for the corresponding ELISA absorbance values). Typically, ELISA absorbance values >0.1 appear non-black in the heat map. Given that the cell wall solvents used do not remove crystalline cellulose, the signals arise from a smaller proportion of the fiber weight (mainly the persistent primary wall) after 22 DPA when secondary wall cellulose synthesis begins [as indicated by material recovered (mg/g AIR) after each extraction step; see bar graphs in Figure 3]. Cell wall polymer epitopes that remained within the cotton fiber primary cell wall from 14 to 35 DPA were indicated by persistent ELISA signals in one or more cell wall extracts, often arising from multiple antibodies within one or more clades (Figure 3; Table S2). The polymer classes in this category included: XG, xylan (recognized by multiple antibodies), ß-1,3-glucan (callose), HG, rhamnogalacturonan-I (RG-I), and arabinogalactan (AG). Of these, only callose was detected within the thick cellulosic secondary wall (Figure 4), as previously shown in TEM studies [35].

The striking difference revealed by Glycome Profiling between the fibers of the two species was the near absence of loosely-bound (oxalate-extractable) XG in the 14 to 21 DPA *Gb* fiber, whereas multiple antibodies in two clades (recognizing fucosylated and non-fucosylated XG epitopes) indicated the presence of these polymers in *Gh* fiber (white box, Figure 3). In the wall fraction extracted next using carbonate, *Gb* fiber also showed less of these epitopes over fewer DPA as compared to *Gh* fiber. Together the data show that loosely-bound XG was a more prominent feature of *Gh* fiber. Note that the ELISA signals arising from many XG antibodies in two clades became similar in both species across all DPA in the 1 M KOH and 4 M KOH fractions, emphasizing that the difference in XG between the *Gh* and *Gb* fiber as detected by Glycome Profiling occurs within the loosely-bound cell wall polymer fraction.

### Immunohistochemistry and Immuno-dot Blotting Revealed the Cellular Context for Differences in the Fiber Cell Wall Polymers of *G. hirsutum* and *G. barbadense*

Immunohistochemical analysis was carried out with a sub-set of the antibodies used in Glycome Profiling on fiber cross-sections of both species at 10 DPA, 17 DPA, 24 DPA, and 35 DPA (Figure 4). Two stages of primary wall or secondary wall deposition are represented by 10 and 17 DPA or 24 and 35 DPA, respectively. At each DPA, successive sections of the same fiber group were labeled with different antibodies, and the labeling with antibodies recognizing callose or HG showed the overall sample morphology. As in Figure 2, these images showed that adjacent fibers from both *Gh* and *Gb* were joined together through 17 DPA when the CFML was still present, whereas they were separated at 24 DPA once the CFML had degraded (Figure 4; see lower magnification views of the fiber cross-sections in Figure S2).

The immunohistochemistry results were consistent with Glycome Profiling in showing the existence of several polymer classes within the primary cell walls that surround the protoplast in both species: XG (labeled with CCRC-M88 in the clade recognizing non-fucosylated XG); ß-1,3-glucan (callose) (labeled with LAMP); HG (labeled with CCRC-M38); RG-I (labeled with CCRC-M35); and AG (labeled with CCRC-M107 and often existing as a side chain of RG-I). XG epitopes were detected in discrete spots corresponding to the bulges in the CFML only in *Gh* fiber, as shown by the asterisks in the panel for labeling with CCRC-M1 that recognizes the fucosylated side chain [a-Fuc-(1,2)-ß-Gal] sometimes found in XG [36]). This result is consistent with previously reported results for *Gh* fiber [3]. A bulge in the CFML in *Gb* fiber was filled with HG (see the arrow in the panel showing 17 DPA fiber labeled with CCRC-M38), but the same bulge did not show any detectable signal with the other probes tested (see the white arrows pointing to the same location in other panels).

A hot water extract of cotton fibers was used to confirm that *Gh* fiber had more XG, including fucosylated and non-fucosylated epitopes, in an unusual loosely-bound cell wall fraction as compared to *Gb* fiber. Hot acidic water is commonly used to straighten cotton fiber prior to hand measurement, and we predicted that this procedure would remove components of the CFML that helps cotton fiber to organize into curved bundles around each seed [3] thereby allowing the fibers to straighten. In preliminary work, 96 antibodies recognizing diverse polysaccharide classes were tested in immuno-dot assays of hot, acidic water extracts of 10, 19, and 24 DPA *Gh* fiber. Twelve antibodies were positive in dot blots of hot water extracts and in TEM immunolabeling of bulges in the CFML (unpublished data). These twelve antibodies recognized both fucosylated and non-fucosylated epitopes of XG and HG with no or relatively low esterification, corresponding to previous results [3]. These preliminary results provided evidence that the CFML polysaccharides were loosely bound and extractable by hot acidic water, which is reasonable given the CFML is an adhesive layer on the outer perimeter of the structural primary wall surrounding the protoplast [3].

**Figure 5:**
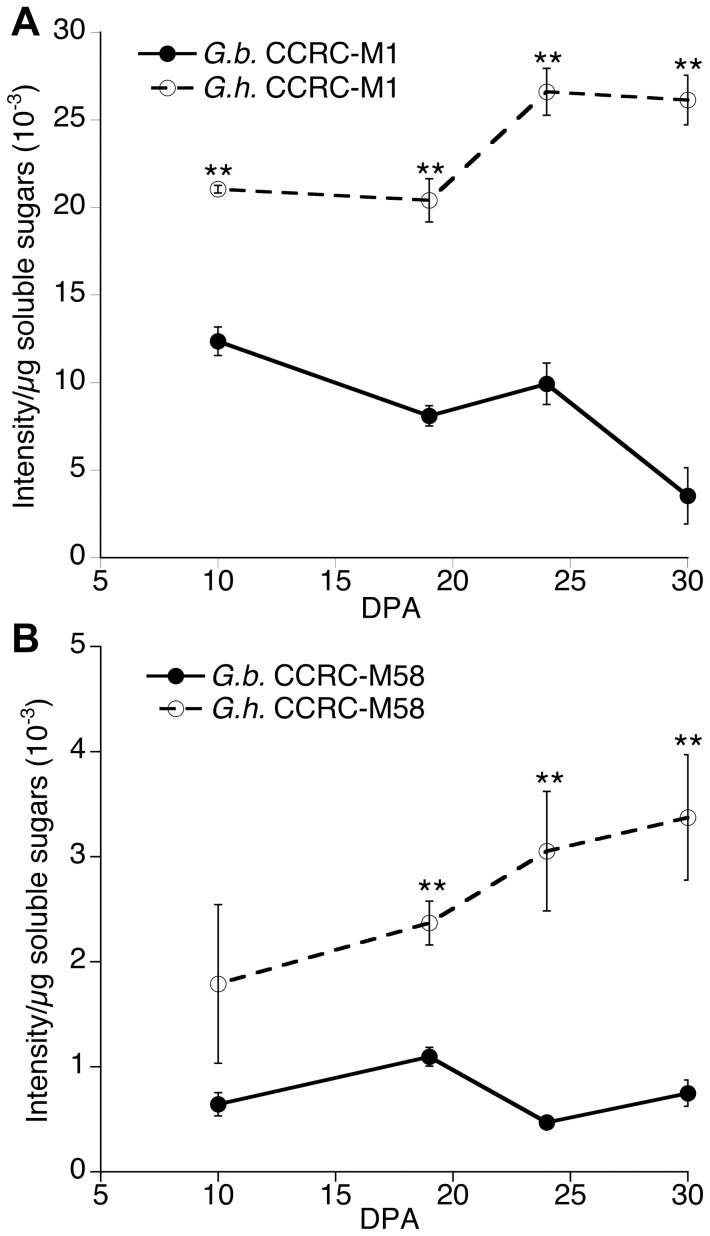
Colorimetric intensity values from immuno-dot-assays of epitopes within cell wall polymers extracted from *G. hirsutum* and *G. barbadense* fiber at 10, 19, 24, and 30 DPA by hot acidic water. Antibody probes used were: (**A**) CCRC-M1 recognizing fucosylated XG epitopes and (**B**) CCRC-M58 recognizing non-fucosylated XG epitopes. Data points are the means (± SE) of 3 biological replications, and asterisks indicate that means from the two species at that DPA are significantly different as determined by T test (**p≤0.01).

The hot acidic water extracts of both *Gh* and *Gb* fiber at 10, 19, 24, and 30 DPA were probed in an immuno-dot assay with CCRC-M1 and CCRC-M58, which recognized fucosylated or non-fucosylated XG epitopes, respectively (Figure 5). Both probes showed that as predicted the hot acidic water extract of 10 and 19 DPA *Gh* fiber (when the CFML was still present) had more XG than *Gb* fiber (Figure 5A and 5B, respectively). The continued detection of high levels of hot water extractable XG in 24 and 30 DPA *Gh* fiber extracts (after the CFML degrades sufficiently to release fibers as individuals) corresponds to the continued detection of XG on the perimeter of *Gh* fiber at 24 and 35 DPA (Figure 4).

### *G. barbadense* had Higher Capacity to Hydrolyze XG Early in Fiber Development

**Figure 6:**
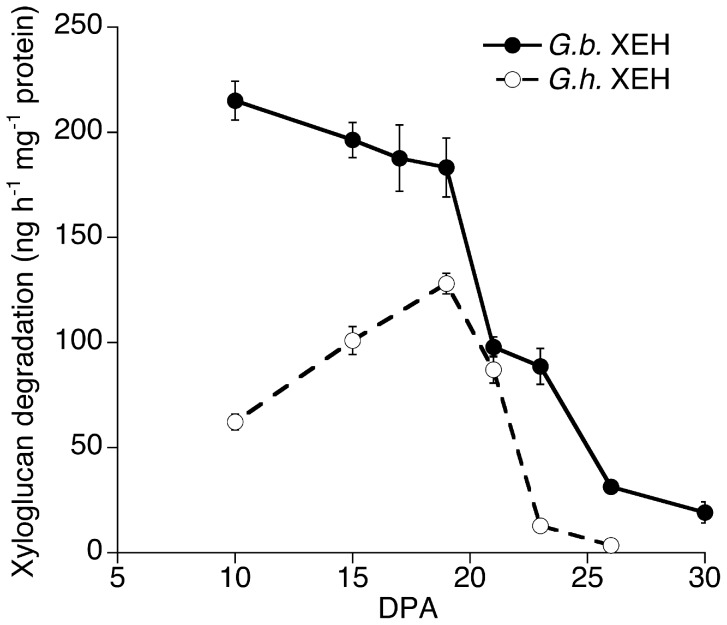
Xyloglucan endo-hydrolase (XEH) activity in developing 10 to 30 DPA fibers of *G. hirsutum* and *G. barbadense*. Data points are the means (± SE) of 4 biological replications. The data for *Gh* are republished from Table SVI of [3] (http://www.plantphysiol.org, Copyright American Society of Plant Biologists).

Xyloglucan endo-hydrolase (XEH) activity was measured in fiber protein extracts to look for evidence that the potential to degrade XG was different in the two species [37]. At 10 to 20 DPA, *Gb* fiber had up to four-fold higher XEH activity compared to *Gh* fiber (Figure 6A), then XEH activity in both species drops sharply after 20 DPA, just before the onset of secondary wall deposition.

## Discussion

Cotton fiber is ideally suited for analysis of cell wall chemistry because it is an isolated single cell that undergoes a prolonged differentiation program including primary wall synthesis, transitional cell wall remodeling, and secondary wall thickening *via* synthesis of nearly 100% cellulose (reviewed in [4]). The current results reflect a comprehensive, inter-specific comparison of cotton fiber cell wall matrix polysaccharide chemistry between 10 and 35 DPA, bolstering the relatively limited knowledge about cotton fiber non-cellulosic polysaccharides [38]. The chemical differences were correlated with cellular differences in cell wall structure and potential for XG hydrolysis using immunohistochemistry and enzyme assays.

The growing conditions used in this study supported good vegetative growth and flowering for both *Gh* and *Gb* plants, as well as production of the superior fiber quality typical of *Gb* grown in the field. The fiber of both species had a thick secondary wall as indicated by high maturity ratios, but the *Gb* fiber was longer, stronger, and finer (less mass per unit length) compared to the *Gh* fiber. Therefore, the moderate temperature and sufficient water and nutrients in the greenhouse supported normal fiber development even for the less adaptable *Gb* cotton plants. Despite their origins from the same (or similar) A and D ancestral genomes, these two allotetraploids have limited potential for interspecific hybridization, and the two species/cultivars we studied are distinct genotypes as indicated by a negligible calculated coefficient of parentage (0.00039; Daryl Bowman, personal communication; [39]). The representative nature of the comparative fiber growth profiles is supported by the similarity of our results to those using other *Gh* and *Gb* cultivars grown in parallel [10], although in this earlier study secondary wall deposition started earlier due to differences in cultivars and/or warmer daytime temperature (∼33 to 36°C; see [40] for growing conditions).

Under the moderate and highly controlled greenhouse conditions used for our experiments, the fiber growth profiles in *Gh* cv. Deltapine 90 and *Gb* cv. Phytogen 800 had remarkable commonalities, given the divergence of the species 1–2 MYA, as well as differences. Commonalities included: (a) similar rate of elongation before 22 DPA, when elongation ended in *Gh*; (b) the presence of a CFML; (c) a developmental shift in primary vs. secondary wall gene expression beginning ∼17 DPA; and (d) onset of secondary wall cellulose synthesis ∼22 DPA, along with the breakdown of the CFML. (See the data on the CFML of *Gh* fiber in [3].) Differences included the expected prolonged period of elongation in *Gb* fiber (resulting in its greater length) and less cellulose deposited per unit length of *Gb* fiber (correlated at least in part with its lower diameter). In addition, the CFML composition was different as will be discussed further below. The highly resolved fiber growth profiles developed in parallel with harvesting fiber samples for analysis allowed strong correlation to be made between fiber cell wall chemistry and particular events in fiber development. These fiber growth profiles are likely to be useful to others when replicated with the same cultivars under the same growing conditions, but variance in any parameter may lead to the need to document the temporal progression of fiber development independently.

### Both *G. hirsutum* and *G. barbadense* Fiber have Primary Walls Including Polymer Classes Typical of other Dicotyledonous Plants

The primary walls of *Gh* fiber contain sugars typical of other primary walls: arabinose, fucose, galactose, glucose, mannose, rhamnose, xylose, glucuronic acid, and galacturonic acid, suggesting the presence of typical primary wall matrix polysaccharides [1], [3]. These constituent sugars are consistent with cell wall matrix polymer classes that were identified in both *Gh* and *Gb* fiber cell walls by use of Glycome Profiling and immunohistochemistry, including XG, xylan, HG, RG-I, and AG (possibly as side chains of RG-I or AG proteins).

The results were consistent with a few studies where isolated cotton fiber wall polymers were chemically characterized. The XG purified from 19 DPA *Gh* fiber with strong alkali had a ß-1,4-glucan backbone and typical XG oligosaccharide motifs [41]. A water-extractable Type II branched AG (with a-1,3-linked galactan backbone and side groups containing arabinose, rhamnose, and glucuronic acid residues) was proposed to exist in the primary wall of 30 to 40 DPA fiber of *G. arboreum*, a diploid A genome cotton species [42]. Atypically as compared to most plant cell walls, the cotton fiber walls in both species at 10 to 24 DPA labeled strongly with the LAMP antibody, which recognizes ß-1,3-glucan (callose) [25]. Most of the callose was strongly bound within the fiber wall as indicated by strong LAMP signals in the alkali extracts during Glycome Profiling. Some callose was immunologically detected within the thickening secondary wall, as observed before in TEM [35]. Other studies also showed the existence of callose in *Gh* and/or *Gb* cotton fiber, although its appearance at the transition stage had been emphasized previously [3], [43]–[44]. The presence of xylans in cotton fibers had not emphasized previously, although they occur in both primary and secondary walls of other dicots [45]–[46]. The Glycome Profiling showed that multiple xylan-directed monoclonal antibodies bound to alkali extracts of 14 to 35 DPA fibers from both species, which is consistent with the binding of other xylan-directed antibodies (LM10 and LM11) to alkali extracts of *Gh* fiber [3]. The specific role of xylan in the cotton fiber cell wall awaits further exploration.

### The CFML was Present but had a Different Composition in *G. barbadense* Fiber

Adjacent *Gb* fibers were joined by a CFML during the first 20 days of fiber development, as previously shown for *Gh* fiber [3]. The CFML was degraded near the onset of secondary wall synthesis in the fiber of both species, despite the fact that *Gb* fiber continued to elongate rapidly after that time. Therefore, the CFML is not directly required for cotton fiber elongation to occur. It remains likely, however, that fiber bundle formation mediated by the CFML facilitates elongation indirectly through organizing >100,000 elongating fibers into efficiently packed tissue-like bundles within a confined space [3].

The polymer composition of the CFML in *Gb* fiber is different compared to *Gh* fiber. Among the probes we tested, only the CCRC-M38 antibody that recognizes HG labeled the CFML bulges between *Gb* fibers, and HG was also found in the CFML of *Gh* fiber (these results and others in [3]). The CCRC-M38 antibody exists in a 16-member clade including other commonly used HG antibodies such as PAM1, JIM5, JIM7, and LM7 [21], [47]. In addition to its use in research on *Arabidopsis thaliana* [48], others used CCRC-M38 to recognize the pectin sheath of 2 DPA *Gh* cotton fibers [49], indicating a continuous role for HG in elongating cotton fiber.

Unlike *Gh* fiber (these results and those in [3]), the CFML of *Gb* fiber lacked XG as detected through immunohistochemistry. This difference is paralleled by differences in the Glycome Profiles fiber cell wall extracts of the two species. The oxalate extract of 14 to 21 DPA *Gh* fiber contained atypical loosely-bound XG, and the diminishment of these epitopes by 24 DPA correlated with CFML degradation just beforehand. In contrast, XG epitopes were largely undetected in the oxalate extracts of *Gb* fiber probed by Glycome Profiling. Similarly, the XG epitopes were much lower in hot acidic water extracts of whole *Gb* fiber probed in immuno-dot-assay. The carbonate fraction of 14 to 17 DPA *Gb* fiber assayed in Glycome Profiling had positive signals with some, but not all, of the antibodies in the non-fucosylated XG clade that worked in *Gh* fiber. Therefore, the molecular structure of the loosely bound XG in the two species is likely somewhat different.

The CFML in *Gb* fiber either contains less XG or contains XG oligomers that were washed out during AIR production for Glycome Profiling and during sample processing for immunohistochemistry. The 70% EtOH washes required for AIR production did not have detectable sugar content (data not shown), but we cannot exclude the possibility that polymers or oligomers were present but were below the detection threshold. The 10 to 19 DPA *Gb* fiber had two- to four-fold higher XEH activity compared to *Gh* fiber, and the additional XEH activity could result in the formation of shorter XG fragments in *Gb* fiber. In future research, it will be worthwhile to isolate and compare the fine structure of XG, as well as potential related oligomers, in developing fiber of the two species. In transgenic *G. hirsutum*, higher capacity to transfer a XG chain end to a different XG polymer (xyloglucan endo-transglycosylase, or XET activity [37]) correlated with longer fiber [50]. In contrast, our results show that the elongation rate was nearly the same in *Gh* and *Gb* fiber during the first 20 DPA when XEH activity was higher in *Gb* fiber. Therefore, increased capacity for XG hydrolysis via XEH activity may affect other aspects of fiber development and quality. Possibly the lower fiber perimeter in *Gb* could be controlled by differences in its cell wall because cell expansion reflects the balance between turgor pressure and cell wall extensibility [33], and this possibility can be tested in further research. Broadly, the Glycome Profiling data provide a rich picture of the types of extractable cell wall matrix polysaccharides that characterize cotton fiber in the two most important commercial species.

## Supporting Information

Figure S1 Effects of interaction between polysaccharides on ELISA absorbance values. Three different polysaccharide preparations were used in this experiment: an ammonium oxalate extract prepared from leaves of *Arabidopsis thaliana* (AO) that is rich in pectic arabinogalactans; a 1 M KOH extract prepared from switchgrass biomass (SG) that is rich in xylans; and a commercially available preparation of non-fucosylated tamarind xyloglucan (XG). Each polysaccharide preparation was assayed in water at concentrations from 1 to 20 µg/mL (**A–C**). Mixing experiments (**D–F**) were carried out by adding two different polysaccharide preparations in inverse concentrations to each other in order to end up with a total carbohydrate concentration of 20 µg/mL in each sample applied to the ELISA plates (50 µL volume). Antibodies used were: CCRC-M16, CCRC-M23, CCRC-M78 and CCRC-M133 recognize pectic arabinogalactan epitopes; CCRC-M150, CCRC-M153 and CCRC-M155 recognize xylan epitopes; and CCRC-M86, CCRC-M96, CCRC-M103 and CCRC-M111 recognize XG epitopes. The increase in the ELISA signal observed with increasing amounts of the AO extract (**A**) was unaffected by the presence of XG **(D)** or the SG extract **(F)**. Likewise, the increase in ELISA signal observed with increasing amounts of the SG extract **(B**) was unaffected by the presence of XG **(E)** or AO extract **(F)**. The increase in ELISA signal from tamarind XG **(C)** was similar in the presence of xylan in the SG extract **(E)**. However, the presence of the pectic arabinogalactan-rich AO extract diminished the ELISA signal for the XG-directed antibodies tested at low concentrations of XG (up to 10 µg/mL). In contrast, at higher XG concentrations, the AO extract had little if any affect **(D)**. These results suggest that at high pectic arabinogalactan concentrations, detection of low concentrations of XG could be compromised. However, the sequential cell wall extraction series, which typically separates most of the pectic arabinogalactans from the hemicelluloses, and the use of multiple antibodies against distinct epitopes within the same polymer class still allows Glycome Profiling to provide an accurate overall picture of cell wall composition.
(DOC)

Figure S2 Overview (lower magnification) fluorescence micrographs of the *Gh* and *Gb* fiber samples shown in Figure 4. The target type of polysaccharide and the antibody used for labeling are shown in the upper left of each panel. In the lower right, the thickening of the secondary wall at 35 DPA is indicated by the fluorescence of Calcofluor White, which stains cellulose and callose. The micrographs for each antibody were taken at the same exposure time. The 25 µm bar in the upper left corner applies to all micrographs.
(DOC)

Table S1 Organization of antibodies into clades and links to additional information about the antibodies used in this study. The groupings of antibodies are based on a hierarchical clustering analysis of all monoclonal antibodies (mAbs) that were screened against a panel of plant polysaccharide preparations (Supplemental Reference [1]). The mAbs are grouped according to the polysaccharides that they predominantly recognize. The majority of listings link to the Wall*Mab*DB plant cell wall mAbs database (http://www.wallmabdb.net), which includes detailed descriptions of each mAb, including immunogen, antibody isotype, epitope structure recognized (where known), supplier information, and related literature references when available.
(DOCX)

Table S2 ELISA absorbance values used to generate the heat map in Figure 3 (XLS file format). The typical value for the water control (absorbance = 0.05) has been subtracted from all values. Values >0.10 are typically detectable in Figure 3, so cells with values <0.10 are shaded light green to facilitate comparison of the two ways of representing the Glycome Profiling data.
(XLS)
