# Supplementary material for: Cotton Fiber Cell Walls of Gossypium hirsutum and Gossypium barbadense Have Differences Related to Loosely-Bound Xyloglucan
Source: PLoS One. 2013 Feb 14;8(2):e56315. doi: 10.1371/journal.pone.0056315 (PMC3572956; doi:10.1371/journal.pone.0056315)
Supplement: Figure S1 — Effects of interaction between polysaccharides on ELISA absorbance values. Three different polysaccharide preparations were used in this experiment: an ammonium oxalate extract prepared from leaves of Arabidopsis thaliana (AO) that is rich in pectic arabinogalactans; a 1 M KOH extract prepared from switchgrass biomass (SG) that is rich in xylans; and a commercially available preparation of non-fucosylated tamarind xyloglucan (XG). Each polysaccharide preparation was assayed in water at concentrations from 1 to 20 µg/mL (A–C). Mixing experiments (D–F) were carried out by adding two different polysaccharide preparations in inverse concentrations to each other in order to end up with a total carbohydrate concentration of 20 µg/mL in each sample applied to the ELISA plates (50 µL volume). Antibodies used were: CCRC-M16, CCRC-M23, CCRC-M78 and CCRC-M133 recognize pectic arabinogalactan epitopes; CCRC-M150, CCRC-M153 and CCRC-M155 recognize xylan epitopes; and CCRC-M86, CCRC-M96, CCRC-M103 and CCRC-M111 recognize XG epitopes. The increase in the ELISA signal observed with increasing amounts of the AO extract (A) was unaffected by the presence of XG (D) or the SG extract (F). Likewise, the increase in ELISA signal observed with increasing amounts of the SG extract (B) was unaffected by the presence of XG (E) or AO extract (F). The increase in ELISA signal from tamarind XG (C) was similar in the presence of xylan in the SG extract (E). However, the presence of the pectic arabinogalactan-rich AO extract diminished the ELISA signal for the XG-directed antibodies tested at low concentrations of XG (up to 10 µg/mL). In contrast, at higher XG concentrations, the AO extract had little if any affect (D). These results suggest that at high pectic arabinogalactan concentrations, detection of low concentrations of XG could be compromised. However, the sequential cell wall extraction series, which typically separates most of the pectic arabinogalactans from the hemicelluloses, and the [file pone.0056315.s001.doc]

Utku Avci, Sivakumar Pattathil, Bir Singh, Virginia L. Brown, Michael G. Hahn,
Candace H. Haigler

The Cotton Fiber Cell Walls of *Gossypium hirsutum* and *Gossypium barbadense* have Differences Related to Loosely-bound Xyloglucan

Figure S1: Effect of mixing of different polysaccharides on the ELISA signals observed with selected mAbs.

| 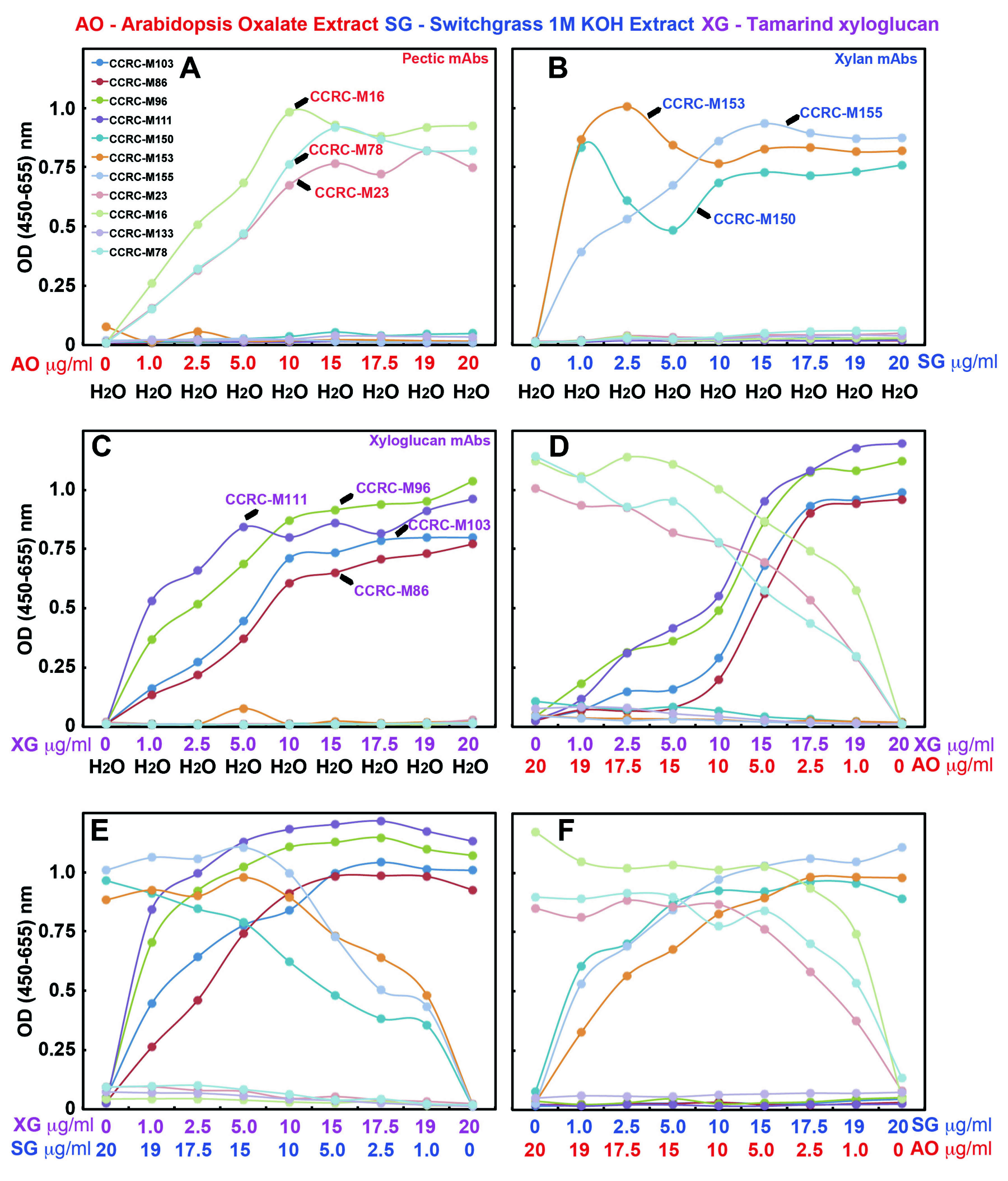 |
| --- |

Figure S1: Effect of mixing of different polysaccharides on the ELISA signals observed with selected mAbs. Three different polysaccharide preparations were used in this experiment: an ammonium oxalate extract prepared from arabidopsis leaves (AO) that is rich in pectic arabinogalactans; a 1 M KOH extract prepared from switchgrass biomass (SG) that is rich in xylans; and a commercially available preparation of non-fucosylated tamarind xyloglucan (XG). Each polysaccharide preparation was assayed in water at concentrations from 1 to 20 μg/mL (**A-C**). Mixing experiments (**D-F**) were carried out by adding two different polysaccharide preparations in inverse concentrations to each other in order to end up with a total carbohydrate concentration of 20 μg/mL in each sample applied to the ELISA plates (50 μL volume). Antibodies used were: CCRC-M16, CCRC-M23, CCRC-M78 and CCRC-M133 recognize pectic arabinogalactan epitopes; CCRC-M150, CCRC-M153 and CCRC-M155 recognize xylan epitopes; and CCRC-M86, CCRC-M96, CCRC-M103 and CCRC-M111 recognize XG epitopes. The increase in the ELISA signal observed with increasing amounts of the AO extract (**A**) was unaffected by the presence of XG **(D)** or the SG extract **(F)**. Likewise, the increase in ELISA signal observed with increasing amounts of the SG extract **(B**) was unaffected by the presence of XG **(E)** or AO extract **(F)**. The increase in ELISA signal from tamarind XG **(C)** was similar in the presence of xylan in the SG extract **(E)**. However, the presence of the pectic arabinogalactan-rich AO extract diminished the ELISA signal for the XG-directed antibodies tested at low concentrations of XG (up to 10 μg/mL). In contrast, at higher XG concentrations, the AO extract had little if any affect **(D)**. These results suggest that at high pectic arabinogalactan concentrations, detection of low concentrations of xyloglucan could be compromised. However, the sequential cell wall extraction series, which typically separates most of the pectic arabinogalactans from the hemicelluloses, and the use of multiple antibodies against distinct epitopes within the same polymer class still allows Glycome Profiling to provide an accurate overall picture of cell wall composition..
