# Supplementary material for: Cotton Fiber Cell Walls of Gossypium hirsutum and Gossypium barbadense Have Differences Related to Loosely-Bound Xyloglucan
Source: PLoS One. 2013 Feb 14;8(2):e56315. doi: 10.1371/journal.pone.0056315 (PMC3572956; doi:10.1371/journal.pone.0056315)
Supplement: Figure S2 — Overview (lower magnification) fluorescence micrographs of the Gh and Gb fiber samples shown in Figure 4. The target type of polysaccharide and the antibody used for labeling are shown in the upper left of each panel. In the lower right, the thickening of the secondary wall at 35 DPA is indicated by the fluorescence of Calcofluor White, which stains cellulose and callose. The micrographs for each antibody were taken at the same exposure time. The 25 µm bar in the upper left corner applies to all micrographs. (DOC) [file pone.0056315.s002.doc]

Utku Avci, Sivakumar Pattathil, Bir Singh, Virginia L. Brown, Michael G. Hahn,
Candace H. Haigler

The Cotton Fiber Cell Walls of *Gossypium hirsutum* and *Gossypium barbadense* have Differences Related to Loosely-bound Xyloglucan

Figure S2: Lower magnification micrographs of immunohistochemistry results shown in Figure 4 for cross-sections of *G. hirsutum* and *G. barbadense* at 10, 17, 24, and 35 DPA.

| 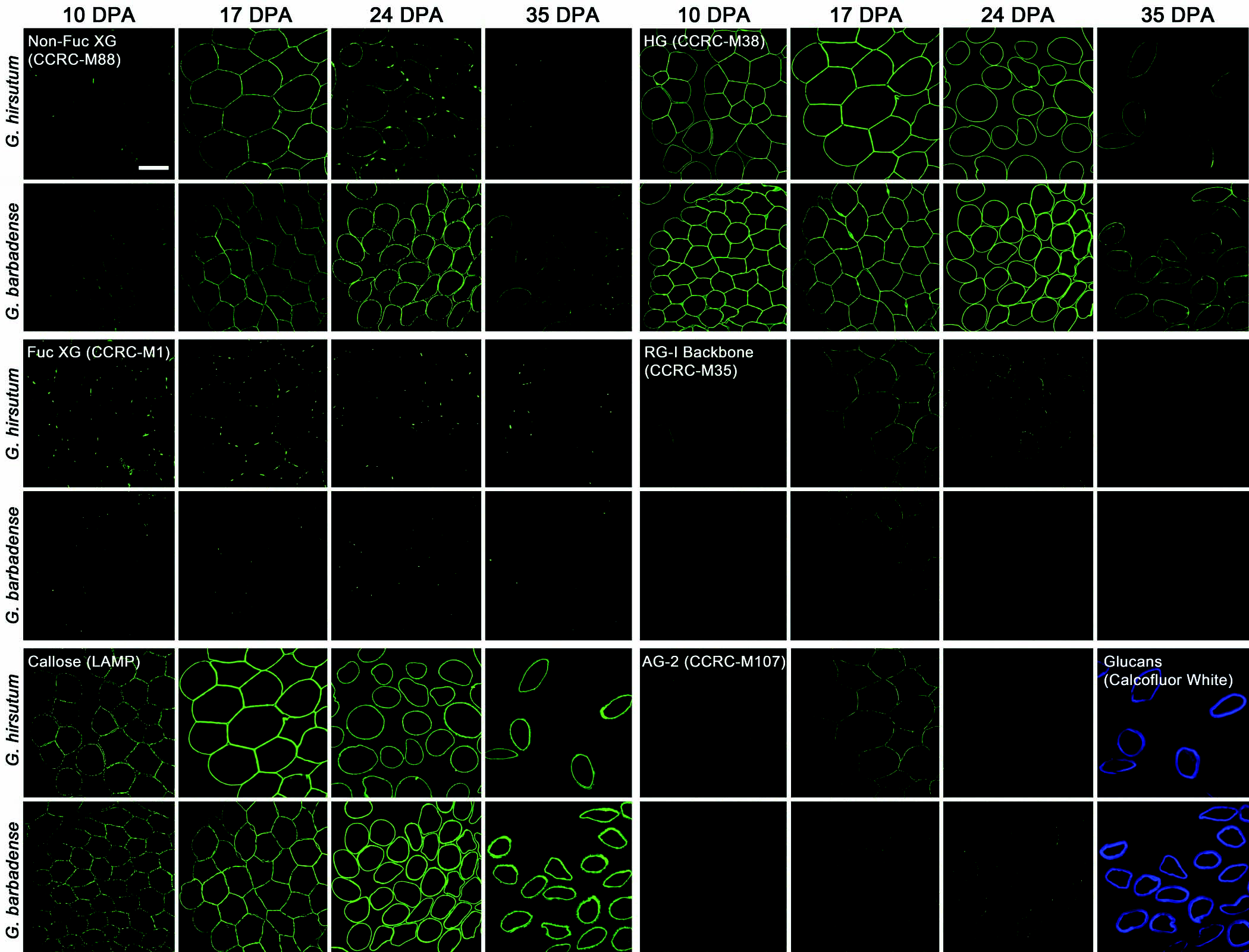 |
| --- |
| Figure S2: Lower magnification micrographs of immunohistochemistry results shown in Figure 4 for cross-sections of *G. hirsutum* and *G. barbadense* at 10, 17, 24, and 35 DPA. The target type of polysaccharide and the antibody used for labeling are shown in the upper left of each panel. In the lower right, the thickening of the secondary wall at 35 DPA is indicated by the fluorescence of Calcofluor White, which stains cellulose and callose. The micrographs for each antibody were taken at the same exposure time. The 25 µm bar in the upper left corner applies to all micrographs. |
