# Supplementary material for: Cotton Fiber Cell Walls of Gossypium hirsutum and Gossypium barbadense Have Differences Related to Loosely-Bound Xyloglucan
Source: PLoS One. 2013 Feb 14;8(2):e56315. doi: 10.1371/journal.pone.0056315 (PMC3572956; doi:10.1371/journal.pone.0056315)
Supplement: Table S1 — Organization of antibodies into clades and links to additional information about the antibodies used in this study. The groupings of antibodies are based on a hierarchical clustering analysis of all monoclonal antibodies (mAbs) that were screened against a panel of plant polysaccharide preparations (Supplemental Reference [1]). The mAbs are grouped according to the polysaccharides that they predominantly recognize. The majority of listings link to the WallMabDB plant cell wall mAbs database (http://www.wallmabdb.net), which includes detailed descriptions of each mAb, including immunogen, antibody isotype, epitope structure recognized (where known), supplier information, and related literature references when available. (DOCX) [file pone.0056315.s003.docx]

Utku Avci, Sivakumar Pattathil, Bir Singh, Virginia L. Brown, Michael G. Hahn,
Candace H. Haigler

The Cotton Fiber Cell Walls of *Gossypium hirsutum* and *Gossypium barbadense* have Differences Related to Loosely-bound Xyloglucan

**Table S1:**  List of plant glycan-directed monoclonal antibodies (mAbs) used in this study.

The groupings of antibodies are based on a hierarchical clustering analysis of all monoclonal antibodies (mAbs) that were screened against a panel of plant polysaccharide preparations (Supplemental Reference [1]). The mAbs are grouped according to the polysaccharides that they predominantly recognize. The majority of listings link to the Wall*Mab*DB plant cell wall mAbs database ([http://www.wallmabdb.net](http://www.wallmabdb.net/)), which includes detailed descriptions of each mAb, including immunogen, antibody isotype, epitope structure recognized (where known), supplier information, and related literature references when available.

**Glycan Group Recognized mAb Name**

| NON-FUC XG  (Non-Fucosylated Xyloglucan) | [CCRC-M54](http://glycomics.ccrc.uga.edu/wall2/jsp/abdetails.jsp?abnumber=147&abname=CCRC-M54) |
| --- | --- |
|  | [CCRC-M48](http://glycomics.ccrc.uga.edu/wall2/jsp/abdetails.jsp?abnumber=77&abname=CCRC-M48) |
|  | [CCRC-M49](http://glycomics.ccrc.uga.edu/wall2/jsp/abdetails.jsp?abnumber=76&abname=CCRC-M49) |
|  | [CCRC-M96](http://glycomics.ccrc.uga.edu/wall2/jsp/abdetails.jsp?abnumber=151&abname=CCRC-M96) |
|  | [CCRC-M50](http://glycomics.ccrc.uga.edu/wall2/jsp/abdetails.jsp?abnumber=143&abname=CCRC-M50) |
|  | [CCRC-M51](http://glycomics.ccrc.uga.edu/wall2/jsp/abdetails.jsp?abnumber=144&abname=CCRC-M51) |
|  | [CCRC-M53](http://glycomics.ccrc.uga.edu/wall2/jsp/abdetails.jsp?abnumber=146&abname=CCRC-M53) |
|  | [CCRC-M100](http://glycomics.ccrc.uga.edu/wall2/jsp/abdetails.jsp?abnumber=114&abname=CCRC-M100) |
|  | [CCRC-M103](http://glycomics.ccrc.uga.edu/wall2/jsp/abdetails.jsp?abnumber=113&abname=CCRC-M103) |
|  | [CCRC-M58](http://glycomics.ccrc.uga.edu/wall2/jsp/abdetails.jsp?abnumber=155&abname=CCRC-M58) |
|  | [CCRC-M86](http://glycomics.ccrc.uga.edu/wall2/jsp/abdetails.jsp?abnumber=157&abname=CCRC-M86) |
|  | [CCRC-M55](http://glycomics.ccrc.uga.edu/wall2/jsp/abdetails.jsp?abnumber=148&abname=CCRC-M55) |
|  | [CCRC-M52](http://glycomics.ccrc.uga.edu/wall2/jsp/abdetails.jsp?abnumber=145&abname=CCRC-M52) |
|  | [CCRC-M99](http://glycomics.ccrc.uga.edu/wall2/jsp/abdetails.jsp?abnumber=152&abname=CCRC-M99) |
|  | [CCRC-M95](http://glycomics.ccrc.uga.edu/wall2/jsp/abdetails.jsp?abnumber=162&abname=CCRC-M95) |
|  | [CCRC-M101](http://glycomics.ccrc.uga.edu/wall2/jsp/abdetails.jsp?abnumber=163&abname=CCRC-M101) |
|  | [CCRC-M104](http://glycomics.ccrc.uga.edu/wall2/jsp/abdetails.jsp?abnumber=164&abname=CCRC-M104) |
|  | [CCRC-M89](http://glycomics.ccrc.uga.edu/wall2/jsp/abdetails.jsp?abnumber=160&abname=CCRC-M89) |
|  | [CCRC-M93](http://glycomics.ccrc.uga.edu/wall2/jsp/abdetails.jsp?abnumber=161&abname=CCRC-M93) |
|  | [CCRC-M87](http://glycomics.ccrc.uga.edu/wall2/jsp/abdetails.jsp?abnumber=158&abname=CCRC-M87) |
|  | [CCRC-M88](http://glycomics.ccrc.uga.edu/wall2/jsp/abdetails.jsp?abnumber=159&abname=CCRC-M88) |
|  | [CCRC-M57](http://glycomics.ccrc.uga.edu/wall2/jsp/abdetails.jsp?abnumber=154&abname=CCRC-M57) |
|  | [CCRC-M90](http://glycomics.ccrc.uga.edu/wall2/jsp/abdetails.jsp?abnumber=156&abname=CCRC-M90) |

|  |  |
| --- | --- |
| FUC XG (Fucosylated Xyloglucan) | [CCRC-M102](http://glycomics.ccrc.uga.edu/wall2/jsp/abdetails.jsp?abnumber=142&abname=CCRC-M102) |
|  | [CCRC-M39](http://glycomics.ccrc.uga.edu/wall2/jsp/abdetails.jsp?abnumber=78&abname=CCRC-M39) |
|  | [CCRC-M106](http://glycomics.ccrc.uga.edu/wall2/jsp/abdetails.jsp?abnumber=112&abname=CCRC-M106) |
|  | [CCRC-M84](http://glycomics.ccrc.uga.edu/wall2/jsp/abdetails.jsp?abnumber=124&abname=CCRC-M84) |
|  | [CCRC-M1](http://glycomics.ccrc.uga.edu/wall2/jsp/abdetails.jsp?abnumber=1&abname=CCRC-M1) |

| Xylan-1/(Xyloglucan) | [CCRC-M111](http://glycomics.ccrc.uga.edu/wall2/jsp/abdetails.jsp?abnumber=168&abname=CCRC-M111) |
| --- | --- |
|  | [CCRC-M108](http://glycomics.ccrc.uga.edu/wall2/jsp/abdetails.jsp?abnumber=149&abname=CCRC-M108) |
|  | [CCRC-M109](http://glycomics.ccrc.uga.edu/wall2/jsp/abdetails.jsp?abnumber=150&abname=CCRC-M109) |

| Xylan-2 | [CCRC-M119](http://glycomics.ccrc.uga.edu/wall2/jsp/abdetails.jsp?abnumber=106&abname=CCRC-M119) |
| --- | --- |
|  | [CCRC-M115](http://glycomics.ccrc.uga.edu/wall2/jsp/abdetails.jsp?abnumber=110&abname=CCRC-M115) |
|  | [CCRC-M110](http://glycomics.ccrc.uga.edu/wall2/jsp/abdetails.jsp?abnumber=167&abname=CCRC-M110) |
|  | [CCRC-M105](http://glycomics.ccrc.uga.edu/wall2/jsp/abdetails.jsp?abnumber=165&abname=CCRC-M105) |
|  | [CCRC-M117](http://glycomics.ccrc.uga.edu/wall2/jsp/abdetails.jsp?abnumber=108&abname=CCRC-M117) |
|  | [CCRC-M113](http://glycomics.ccrc.uga.edu/wall2/jsp/abdetails.jsp?abnumber=171&abname=CCRC-M113) |
|  | [CCRC-M120](http://glycomics.ccrc.uga.edu/wall2/jsp/abdetails.jsp?abnumber=105&abname=CCRC-M120) |
|  | [CCRC-M118](http://glycomics.ccrc.uga.edu/wall2/jsp/abdetails.jsp?abnumber=107&abname=CCRC-M118) |
|  | [CCRC-M116](http://glycomics.ccrc.uga.edu/wall2/jsp/abdetails.jsp?abnumber=109&abname=CCRC-M116) |
|  | [CCRC-M114](http://glycomics.ccrc.uga.edu/wall2/jsp/abdetails.jsp?abnumber=111&abname=CCRC-M114) |
|  | CCRC-M154 |
|  | CCRC-M150 |
|  |  |
| Xylan-3 | CCRC-M160 |
|  | [CCRC-M137](http://glycomics.ccrc.uga.edu/wall2/jsp/abdetails.jsp?abnumber=173&abname=CCRC-M137) |
|  | CCRC-M152 |
|  | CCRC-M149 |
|  | CCRC-M144 |
|  | CCRC-M146 |
|  | CCRC-M145 |
|  | CCRC-M155 |
|  |  |
| Xylan-4 | CCRC-M153 |
|  | CCRC-M151 |
|  | CCRC-M148 |
|  | [CCRC-M140](http://glycomics.ccrc.uga.edu/wall2/jsp/abdetails.jsp?abnumber=176&abname=CCRC-M140) |
|  | [CCRC-M139](http://glycomics.ccrc.uga.edu/wall2/jsp/abdetails.jsp?abnumber=175&abname=CCRC-M139) |
|  | [CCRC-M138](http://glycomics.ccrc.uga.edu/wall2/jsp/abdetails.jsp?abnumber=174&abname=CCRC-M138) |
|  |  |
| Acetylated mannan | CCRC-M170 |
|  |  |
| β-Glucan | [LAMP](http://glycomics.ccrc.uga.edu/wall2/jsp/abdetails.jsp?abnumber=47&abname=LAMP2H12H7) |
|  |  |
| Galactomannan | [CCRC-M75](http://glycomics.ccrc.uga.edu/wall2/jsp/abdetails.jsp?abnumber=133&abname=CCRC-M75) |
|  | [CCRC-M70](http://glycomics.ccrc.uga.edu/wall2/jsp/abdetails.jsp?abnumber=61&abname=CCRC-M70) |
|  | [CCRC-M74](http://glycomics.ccrc.uga.edu/wall2/jsp/abdetails.jsp?abnumber=134&abname=CCRC-M74) |

| HG BACKBONE  (Homogalacturonan Backbone) | [CCRC-M131](http://glycomics.ccrc.uga.edu/wall2/jsp/abdetails.jsp?abnumber=181&abname=CCRC-M131) |
| --- | --- |
|  | [CCRC-M38](http://glycomics.ccrc.uga.edu/wall2/jsp/abdetails.jsp?abnumber=45&abname=CCRC-M38) |
|  | [JIM5](http://glycomics.ccrc.uga.edu/wall2/jsp/abdetails.jsp?abnumber=14&abname=JIM5) |
|  | [JIM136](http://glycomics.ccrc.uga.edu/wall2/jsp/abdetails.jsp?abnumber=57&abname=JIM136) |
|  | [JIM7](http://glycomics.ccrc.uga.edu/wall2/jsp/abdetails.jsp?abnumber=13&abname=JIM7) |
|  | [CCRC-M34](http://glycomics.ccrc.uga.edu/wall2/jsp/abdetails.jsp?abnumber=36&abname=CCRC-M34) |
|  |  |
| RG-I BACKBONE  (Rhamnogalacturonan-I Backbone) | [CCRC-M69](http://glycomics.ccrc.uga.edu/wall2/jsp/abdetails.jsp?abnumber=172&abname=CCRC-M69) |
|  | [CCRC-M35](http://glycomics.ccrc.uga.edu/wall2/jsp/abdetails.jsp?abnumber=66&abname=CCRC-M35) |
|  | [CCRC-M36](http://glycomics.ccrc.uga.edu/wall2/jsp/abdetails.jsp?abnumber=37&abname=CCRC-M36) |
|  | [CCRC-M14](http://glycomics.ccrc.uga.edu/wall2/jsp/abdetails.jsp?abnumber=67&abname=CCRC-M14) |
|  | [CCRC-M129](http://glycomics.ccrc.uga.edu/wall2/jsp/abdetails.jsp?abnumber=104&abname=CCRC-M129) |
|  | [CCRC-M72](http://glycomics.ccrc.uga.edu/wall2/jsp/abdetails.jsp?abnumber=135&abname=CCRC-M72) |
|  |  |
| Linseed Mucilage Rhamnogalacturonan-I | [CCRC-M40](http://glycomics.ccrc.uga.edu/wall2/jsp/abdetails.jsp?abnumber=83&abname=CCRC-M40) |
|  |  |
| Physcomitrella Pectin | [CCRC-M98](http://glycomics.ccrc.uga.edu/wall2/jsp/abdetails.jsp?abnumber=115&abname=CCRC-M98) |
|  | [CCRC-M94](http://glycomics.ccrc.uga.edu/wall2/jsp/abdetails.jsp?abnumber=118&abname=CCRC-M94) |
|  |  |
| RG-Ia  (Rhamnogalacturonan-Ia) | [CCRC-M5](http://glycomics.ccrc.uga.edu/wall2/jsp/abdetails.jsp?abnumber=81&abname=CCRC-M5) |
|  | [CCRC-M2](http://glycomics.ccrc.uga.edu/wall2/jsp/abdetails.jsp?abnumber=8&abname=CCRC-M2) |
|  |  |
| RG-Ib  (Rhamnogalacturonan-Ib) | [CCRC-M23](http://glycomics.ccrc.uga.edu/wall2/jsp/abdetails.jsp?abnumber=92&abname=CCRC-M23) |
|  | [CCRC-M17](http://glycomics.ccrc.uga.edu/wall2/jsp/abdetails.jsp?abnumber=74&abname=CCRC-M17) |
|  | [CCRC-M19](http://glycomics.ccrc.uga.edu/wall2/jsp/abdetails.jsp?abnumber=0&abname=CCRC-M19) |
|  | [CCRC-M18](http://glycomics.ccrc.uga.edu/wall2/jsp/abdetails.jsp?abnumber=0&abname=CCRC-M18) |
|  | [CCRC-M56](http://glycomics.ccrc.uga.edu/wall2/jsp/abdetails.jsp?abnumber=141&abname=CCRC-M56) |
|  | [CCRC-M16](http://glycomics.ccrc.uga.edu/wall2/jsp/abdetails.jsp?abnumber=73&abname=CCRC-M16) |

|  |  |
| --- | --- |
| RG-Ic  (Rhamnogalacturonan-Ic) | [JIM137](http://glycomics.ccrc.uga.edu/wall2/jsp/abdetails.jsp?abnumber=58&abname=JIM137) |
|  | [JIM101](http://glycomics.ccrc.uga.edu/wall2/jsp/abdetails.jsp?abnumber=55&abname=JIM101) |
|  | [CCRC-M61](http://glycomics.ccrc.uga.edu/wall2/jsp/abdetails.jsp?abnumber=138&abname=CCRC-M61) |
|  | [CCRC-M30](http://glycomics.ccrc.uga.edu/wall2/jsp/abdetails.jsp?abnumber=33&abname=CCRC-M30) |

| RG-I/AG  (Rhamnogalacturonan-I/ Arabinogalactan) | [CCRC-M60](http://glycomics.ccrc.uga.edu/wall2/jsp/abdetails.jsp?abnumber=139&abname=CCRC-M60) |
| --- | --- |
|  | [CCRC-M41](http://glycomics.ccrc.uga.edu/wall2/jsp/abdetails.jsp?abnumber=82&abname=CCRC-M41) |
|  | [CCRC-M80](http://glycomics.ccrc.uga.edu/wall2/jsp/abdetails.jsp?abnumber=128&abname=CCRC-M80) |
|  | [CCRC-M79](http://glycomics.ccrc.uga.edu/wall2/jsp/abdetails.jsp?abnumber=129&abname=CCRC-M79) |
|  | [CCRC-M44](http://glycomics.ccrc.uga.edu/wall2/jsp/abdetails.jsp?abnumber=68&abname=CCRC-M44) |
|  | [CCRC-M33](http://glycomics.ccrc.uga.edu/wall2/jsp/abdetails.jsp?abnumber=75&abname=CCRC-M33) |
|  | [CCRC-M32](http://glycomics.ccrc.uga.edu/wall2/jsp/abdetails.jsp?abnumber=35&abname=CCRC-M32) |
|  | [CCRC-M13](http://glycomics.ccrc.uga.edu/wall2/jsp/abdetails.jsp?abnumber=43&abname=CCRC-M13) |
|  | [CCRC-M42](http://glycomics.ccrc.uga.edu/wall2/jsp/abdetails.jsp?abnumber=86&abname=CCRC-M42) |
|  | [CCRC-M24](http://glycomics.ccrc.uga.edu/wall2/jsp/abdetails.jsp?abnumber=93&abname=CCRC-M24) |
|  | [CCRC-M12](http://glycomics.ccrc.uga.edu/wall2/jsp/abdetails.jsp?abnumber=71&abname=CCRC-M12) |
|  | [CCRC-M7](http://glycomics.ccrc.uga.edu/wall2/jsp/abdetails.jsp?abnumber=3&abname=CCRC-M7) |
|  | [CCRC-M77](http://glycomics.ccrc.uga.edu/wall2/jsp/abdetails.jsp?abnumber=131&abname=CCRC-M77) |
|  | [CCRC-M25](http://glycomics.ccrc.uga.edu/wall2/jsp/abdetails.jsp?abnumber=84&abname=CCRC-M25) |
|  | [CCRC-M9](http://glycomics.ccrc.uga.edu/wall2/jsp/abdetails.jsp?abnumber=69&abname=CCRC-M9) |
|  | [CCRC-M128](http://glycomics.ccrc.uga.edu/wall2/jsp/abdetails.jsp?abnumber=183&abname=CCRC-M128) |
|  | [CCRC-M126](http://glycomics.ccrc.uga.edu/wall2/jsp/abdetails.jsp?abnumber=184&abname=CCRC-M126) |
|  | [CCRC-M125](http://glycomics.ccrc.uga.edu/wall2/jsp/abdetails.jsp?abnumber=185&abname=CCRC-M125) |
|  | [CCRC-M123](http://glycomics.ccrc.uga.edu/wall2/jsp/abdetails.jsp?abnumber=187&abname=CCRC-M123) |
|  | [CCRC-M122](http://glycomics.ccrc.uga.edu/wall2/jsp/abdetails.jsp?abnumber=188&abname=CCRC-M122) |
|  | [CCRC-M121](http://glycomics.ccrc.uga.edu/wall2/jsp/abdetails.jsp?abnumber=189&abname=CCRC-M121) |
|  | [CCRC-M112](http://glycomics.ccrc.uga.edu/wall2/jsp/abdetails.jsp?abnumber=169&abname=CCRC-M112) |
|  | [CCRC-M21](http://glycomics.ccrc.uga.edu/wall2/jsp/abdetails.jsp?abnumber=88&abname=CCRC-M21) |
|  | [JIM131](http://glycomics.ccrc.uga.edu/wall2/jsp/abdetails.jsp?abnumber=94&abname=JIM131) |
|  | [CCRC-M22](http://glycomics.ccrc.uga.edu/wall2/jsp/abdetails.jsp?abnumber=46&abname=CCRC-M22) |
|  | [JIM132](http://glycomics.ccrc.uga.edu/wall2/jsp/abdetails.jsp?abnumber=56&abname=JIM132) |
|  | [JIM1](http://glycomics.ccrc.uga.edu/wall2/jsp/abdetails.jsp?abnumber=79&abname=JIM1) |
|  | [CCRC-M15](http://glycomics.ccrc.uga.edu/wall2/jsp/abdetails.jsp?abnumber=72&abname=CCRC-M15) |
|  | [CCRC-M8](http://glycomics.ccrc.uga.edu/wall2/jsp/abdetails.jsp?abnumber=29&abname=CCRC-M8) |
|  | [MH4.3E5](http://glycomics.ccrc.uga.edu/wall2/jsp/abdetails.jsp?abnumber=100&abname=MH4.3E5) |
|  | [JIM16](http://glycomics.ccrc.uga.edu/wall2/jsp/abdetails.jsp?abnumber=62&abname=JIM16) |

|  |  |
| --- | --- |
| AG-1  (Arabinogalactan-1) | [JIM93](http://glycomics.ccrc.uga.edu/wall2/jsp/abdetails.jsp?abnumber=117&abname=JIM93) |
|  | [JIM94](http://glycomics.ccrc.uga.edu/wall2/jsp/abdetails.jsp?abnumber=95&abname=JIM94) |
|  | [JIM11](http://glycomics.ccrc.uga.edu/wall2/jsp/abdetails.jsp?abnumber=41&abname=JIM11) |
|  | [MAC204](http://glycomics.ccrc.uga.edu/wall2/jsp/abdetails.jsp?abnumber=23&abname=MAC204) |
|  | [JIM20](http://glycomics.ccrc.uga.edu/wall2/jsp/abdetails.jsp?abnumber=91&abname=JIM20) |

| AG-2  (Arabinogalactan-2) | [JIM14](http://glycomics.ccrc.uga.edu/wall2/jsp/abdetails.jsp?abnumber=31&abname=JIM14) |
| --- | --- |
|  | [MAC207](http://glycomics.ccrc.uga.edu/wall2/jsp/abdetails.jsp?abnumber=22&abname=MAC207) |
|  | [JIM19](http://glycomics.ccrc.uga.edu/wall2/jsp/abdetails.jsp?abnumber=44&abname=JIM19) |
|  | [JIM12](http://glycomics.ccrc.uga.edu/wall2/jsp/abdetails.jsp?abnumber=191&abname=JIM12) |
|  | [CCRC-M133](http://glycomics.ccrc.uga.edu/wall2/jsp/abdetails.jsp?abnumber=96&abname=JIM133) |
|  | [CCRC-M107](http://glycomics.ccrc.uga.edu/wall2/jsp/abdetails.jsp?abnumber=166&abname=CCRC-M107) |
|  |  |
| AG-3  (Arabinogalactan-3) | [JIM4](http://glycomics.ccrc.uga.edu/wall2/jsp/abdetails.jsp?abnumber=40&abname=JIM4) |
|  | [CCRC-M31](http://glycomics.ccrc.uga.edu/wall2/jsp/abdetails.jsp?abnumber=34&abname=CCRC-M31) |
|  | [JIM17](http://glycomics.ccrc.uga.edu/wall2/jsp/abdetails.jsp?abnumber=39&abname=JIM17) |
|  | [CCRC-M26](http://glycomics.ccrc.uga.edu/wall2/jsp/abdetails.jsp?abnumber=85&abname=CCRC-M26) |
|  | [JIM15](http://glycomics.ccrc.uga.edu/wall2/jsp/abdetails.jsp?abnumber=32&abname=JIM15) |
|  | [JIM8](http://glycomics.ccrc.uga.edu/wall2/jsp/abdetails.jsp?abnumber=80&abname=JIM8) |
|  | [CCRC-M85](http://glycomics.ccrc.uga.edu/wall2/jsp/abdetails.jsp?abnumber=121&abname=CCRC-M85) |
|  | [CCRC-M81](http://glycomics.ccrc.uga.edu/wall2/jsp/abdetails.jsp?abnumber=127&abname=CCRC-M81) |
|  | [MAC266](http://glycomics.ccrc.uga.edu/wall2/jsp/abdetails.jsp?abnumber=98&abname=MAC266) |
|  | [PN 16.4B4](http://glycomics.ccrc.uga.edu/wall2/jsp/abdetails.jsp?abnumber=11&abname=PN%2016.4B4) |

|  |  |
| --- | --- |
| AG-4  (Arabinogalactan-4) | [JIM133](http://glycomics.ccrc.uga.edu/wall2/jsp/abdetails.jsp?abnumber=96&abname=JIM133) |
|  | [JIM13](http://glycomics.ccrc.uga.edu/wall2/jsp/abdetails.jsp?abnumber=30&abname=JIM13) |
|  | [CCRC-M92](http://glycomics.ccrc.uga.edu/wall2/jsp/abdetails.jsp?abnumber=119&abname=CCRC-M92) |
|  | [CCRC-M91](http://glycomics.ccrc.uga.edu/wall2/jsp/abdetails.jsp?abnumber=120&abname=CCRC-M91) |
|  | [CCRC-M78](http://glycomics.ccrc.uga.edu/wall2/jsp/abdetails.jsp?abnumber=130&abname=CCRC-M78) |
|  |  |
| Unidentified | [MAC265](http://glycomics.ccrc.uga.edu/wall2/jsp/abdetails.jsp?abnumber=97&abname=MAC265) |
|  | [CCRC-M97](http://glycomics.ccrc.uga.edu/wall2/jsp/abdetails.jsp?abnumber=116&abname=CCRC-M97) |
|  |  |

**Supplemental Reference:**

1. Pattathil S, Avci U, Baldwin D, Alton GS, McGill JA, et al. (2010) A comprehensive toolkit of plant cell wall glycan-directed monoclonal antibodies. Plant Physiol 153: 514-525.
